# Uniaxial Drawing of Graphene-PVA Nanocomposites: Improvement in Mechanical Characteristics via Strain-Induced Exfoliation of Graphene

**DOI:** 10.1186/s11671-016-1595-2

**Published:** 2016-08-24

**Authors:** Rahim Jan, Amir Habib, Muhammad Aftab Akram, Tanveer-ul-Haq Zia, Ahmad Nawaz Khan

**Affiliations:** School of Chemical and Materials Engineering, National University of Sciences and Technology, H-12 Campus, Islamabad, Pakistan

**Keywords:** Liquid phase exfoliation, Polymer grafting, Strain-induced exfoliation, Graphene, Nanocomposites

## Abstract

Polyvinyl alcohol (PVA)-stabilized graphene nanosheets (GNS) of lateral dimension (*L*) ~1 μm are obtained via liquid phase exfoliation technique to prepare its composites in the PVA matrix. These composites show low levels of reinforcements due to poor alignment of GNS within the matrix as predicted by the modified Halpin-Tsai model. Drawing these composites up to 200 % strain, a significant improvement in mechanical properties is observed. Maximum values for Young’s modulus and strength are ~×4 and ~×2 higher respectively than that of neat PVA. Moreover, the rate of increase of the modulus with GNS volume fraction is up to 700 GPa, higher than the values predicted using the Halpin-Tsai theory. However, alignment along with strain-induced de-aggregation of GNS within composites accounts well for the obtained results as confirmed by X-ray diffraction (XRD) characterization.

## Background

Polymer nanocomposites (PNCs) present a unique scope for many technological applications. It is an ever growing field due to their ease of processing and range of properties achieved with the addition of various nano-fillers [1]. Among the broad range of available nano-fillers, graphene is a single layer (~0.35 nm thick) of carbon atoms arranged in a two-dimensional (2D) honeycomb crystal lattice [2]. The multi-faceted properties such as highest mechanical strength, very high electron mobility, good thermal conductivity, and high specific surface area possessed by graphene make it an ideal choice to be employed in PNCs [3, 4]. Graphene can impart the reinforcement near to theoretical acclaimed results of its exceedingly high modulus (~1 TPa) and strength (~130 GPa) when used as a nano-filler in polymers [5]. The degree of reinforcement depends on the lateral size, dispersion state and orientation of the nano-filler within, and its bonding to the matrix. Liquid phase exfoliation along with the centrifugation process can be utilized for size-selected graphene dispersions in large quantities [6, 7] so that large aspect ratio graphene nanosheets (GNS) can readily be dispersed in polymer solutions. It has been an established fact that the nano-filler extent of dispersion and alignment directly affects the mechanical properties of the composites [8, 9]. Therefore, to avoid aggregation and have better dispersion, chemical functionalization of nano-filler has effectively been employed [10]; however, it somehow compromises the quality of graphene. Another approach utilized successfully for the reinforcement purposes is to stabilizing GNS with the polymer also termed as polymer grafting to GNS [5]. Ultrasonication can create defective sites both in graphene and polymers which helps in the formation of polymer-functionalized/stabilized graphene. This polymer-stabilized graphene can readily be dispersed in the same polymer to form composites [11].

The orientation effect is induced in the composites through the post-treatment technique of drawing. This technique is normally utilized during polymer-fiber fabrication process and 200–500 % strains are applied depending on the polymer system. Drawing causes chain alignment along with an improvement in dispersion state of nano-filler inside the matrix; thus, increasing crystallinity and mechanical characteristics such as strength and modulus enhancement level can be raised by a factor of 8/3 [12, 13]. This approach has also been applied to composites with 2D fillers; although fewer papers are available, most of them are related to clay-filled composites [14–17]. Only two reports of drawing of composites with 2D materials other than clay have been found; (1) containing graphene oxide and (2) hexagonal boron nitride (hBN) nanosheets [18, 19]. Surprisingly; no work is done about drawing of graphene (pristine)-filled polymer composite. Our earlier work with the hBN-PVC system [19] has confirmed the polymer chain alignment of the filler in the direction of drawing along with strain-induced exfoliation and de-aggregation of hBN. It is worthwhile here to examine the effects of drawing in GNS-filled PVA polymer nanocomposites. The use of such composite will be helpful in determining the level of strain-induced exfoliation and de-lamination via XRD technique as both the graphene and PVA have their own characteristic diffraction peaks. Here in this work, we have prepared composites of PVA filled with GNS and observed relatively low extent of reinforcement level before drawing probably due to the random and misaligned layers of GNS. After drawing to 200 % strain, these composites showed a significant enhancement in mechanical properties owing to the orientation and strain-induced exfoliation of GNS inside the PVA matrix.

## Methods

**Fig. 1:**
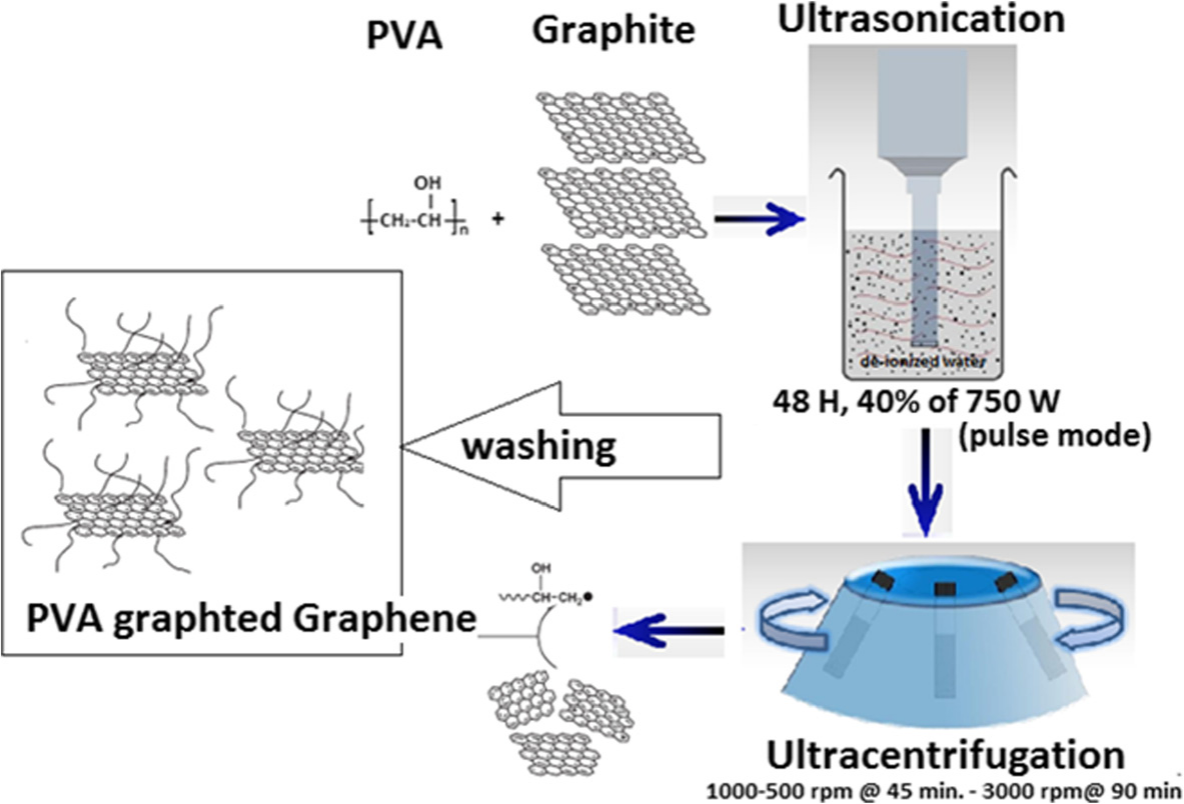
Schematic diagram for polymer grafting of graphene (PVA-stabilized graphene nanosheets). Adapted from [10]. Photograph, taken immediately after adding GNS dispersion into the PVA solution, clearly shows the PVA grafting to GNS effect

PVA (polyvinyl alcohol; Baker Lot No. L06611, molecular weight = 77,000 to 79,000 g/mol) is suspended in 100-ml deionized water (H_2_O) in a round bottom flask (30 mg/ml). Graphite flakes (Branwell Graphite Limited, grade RFL 99.5) are added (6 g) to the PVA/H_2_O solution and sonicated for 48 h using a flat head probe sonic tip. The final exfoliated solution of PVA/GNS/H_2_O is centrifuged at 1000 rpm for 45 min. The supernatant is transferred out to collect the exfoliated material of a uniform size. The sediment is again centrifuged at 500 rpm for 45 min and the supernatant is taken out. The 500-rpm supernatant is then centrifuged at 3000 rpm for 90 min to remove the smaller size exfoliated flakes. The sediments of this sample are filtered through 0.47-μm nylon membranes by adding more deionized water to it. Deionized water is used to remove out the excessive PVA which might be degraded during the exfoliation process. The sample is dried overnight in an oven and then a stock solution (6 mg/ml) in deionized water is prepared for composite solutions. The schematic diagram (Fig. 1) is shown for the synthesis mechanism of polymer-grafted graphene (PVA-stabilized graphene). The various composites of graphene dispersions (0, 0.0001, 0.0003, 0.0006, and 0.006) *V*_*f*_ GNS are added to PVA/deionized water solution, bath sonicated for 4 h, and drop cast into Teflon moulds. The moulds are placed in a vacuum oven at 60 °C for 24 h to dry out. Composite films are peeled off and kept in sealed polythene bags to protect from environmental effects; this set of composites is called as-prepared composites.

Another set of composites (same GNS volume fraction range) is kept at room temperature for 24 h so that the composites are not fully dried, and some solvent is trapped inside to strain the composite films up to 200–350 % termed as drawn composites. Drawing process is performed using a Shimadzu tensile tester at a strain rate of 5 mm/min. After drawing, the composite set is then kept in the vacuum oven at 60 °C overnight to completely remove the solvent.

## Results and Discussion

### Dimension Morphology of GNS and GNS-PVA Composites

The main course of this work lies in the liquid phase exfoliation of graphene in PVA/H_2_O deionized water solution by utilizing ultrasonic irradiation and its size selection by utilizing centrifugation process. It has been shown by Shen et al. [11] that the ultrasonic irradiation of graphene in PVA/H_2_O solution can cause a substantial quantity of defects on the surface of GNS during cavitation and collapse process. These defects can act as reactive sites for the PVA chain radicals produced due to the degradation of PVA solution via ultrasonication, providing polymer-stabilized graphene [20–22]. The polymer-stabilized GNS dispersions are ideal for further processing of polymer nanocomposites. The dimensions of GNS play an important role in improving the mechanical properties and thus are to be assessed carefully. Raman spectroscopy is possibly one of the most common characterization methods used for graphene, which can provide information about various properties including nanosheet mean length and number of layers [23–26]. It has been an established fact that the ratio of D-peak and G-peak intensities (*I*_*D*_/*I*_*G*_) gives a quantitative estimation of the mean length value < L > of nanosheets as shown below [27]:

1 $$ <L > =\frac{0.094}{{\left({I}_D/{I}_G\right)}_{\mathrm{GNS}}-{\left({I}_D/{I}_G\right)}_{\mathrm{Graphite}}} $$

**Fig. 2:**
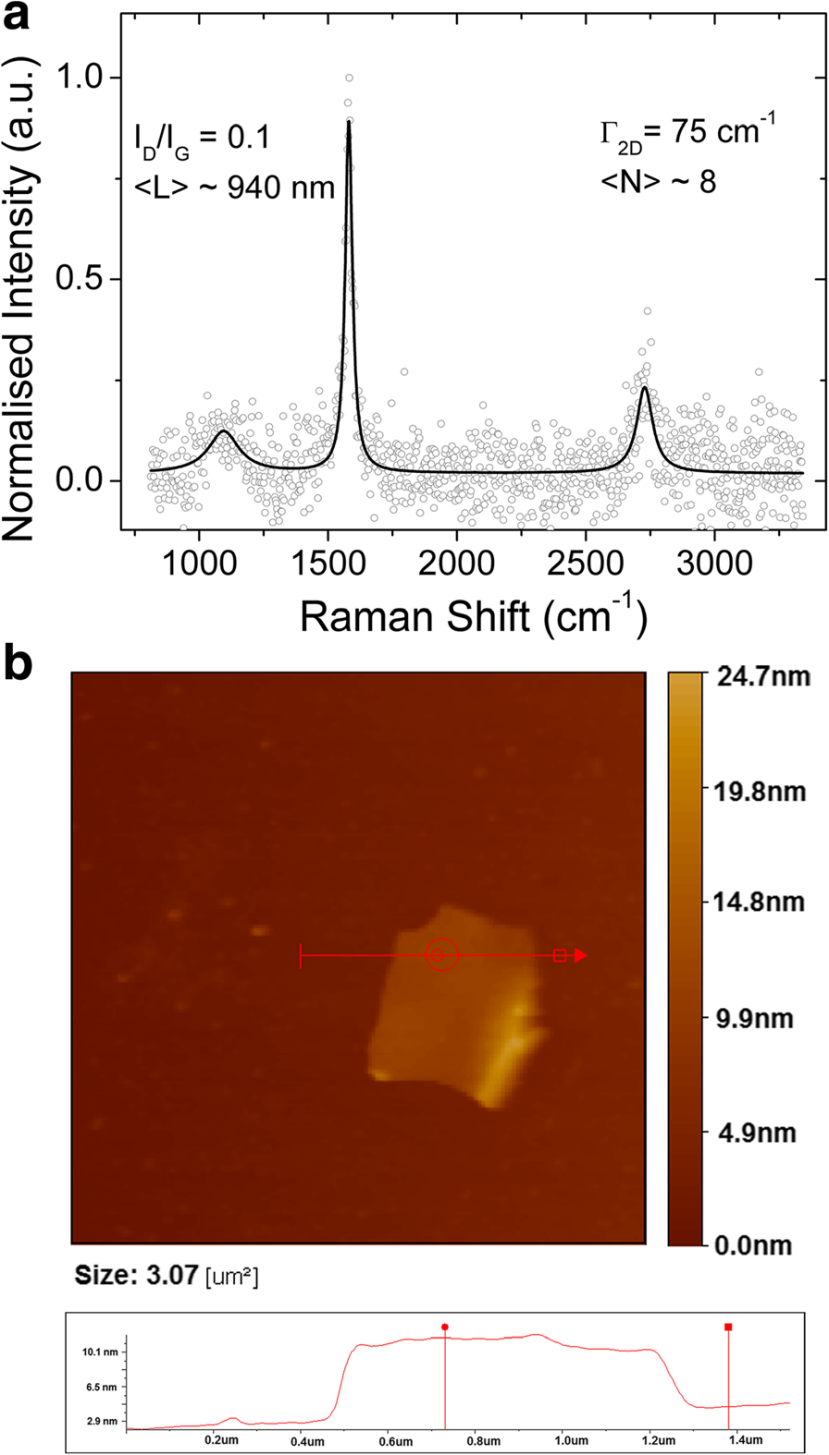
**a** Raman spectroscopy and **b** atomic force microscopy (representative image with a step height profile) characterization for the estimation of mean nanosheet length < L > ~1 μm and number of layers per flake < N > ~6–8

< L > is expressed in micrometers with the estimated error to be ~20 %. Assuming the parent graphite is free of defects, the contribution of D-peak is negligible as compared to G-peak for graphite. Raman spectrum on GNS is performed by using the Dongwoo Optron setup with the laser excitation wavelength of 532 nm and power of 40 mW. Figure 2a shows D-, G-, and 2D peaks, with the intensity normalized to the G-peak of the Raman spectra of the GNS dried onto the glass slide. The solid line is not the actual spectrum but a fit to 3 Lorentzian. As evident from the D- and G-peaks, the *I*_*D*_/*I*_*G*_ value is around 0.1 and the estimated mean length value < L > of GNS is found to be ~0.94 μm. In the recent work of Coleman et al. [27], few metrics have been developed for assessing the number of layers “*N*” for GNS on the basis of intensity and full width half maximum (FWHM) values of 2D band. The problem with the intensity-based metrics is that it requires a fixed wavenumber which makes the resultant value sensitive to any instrumental shifts in position of spectra. The FWHM-related metric is reasonable for the present work. The number of layers per nanosheet can be calculated by the following equation:

2 $$ <N > =\frac{\varGamma_{2\mathrm{D}}-50.3}{3.05} $$

Γ_2D_ is the FWHM value of the 2D band which is around 74 cm^−1^ as shown in Fig. 2a. The number of layers per nanosheet, < N >, are around 8 according to Eq. (2).

**Fig. 3:**
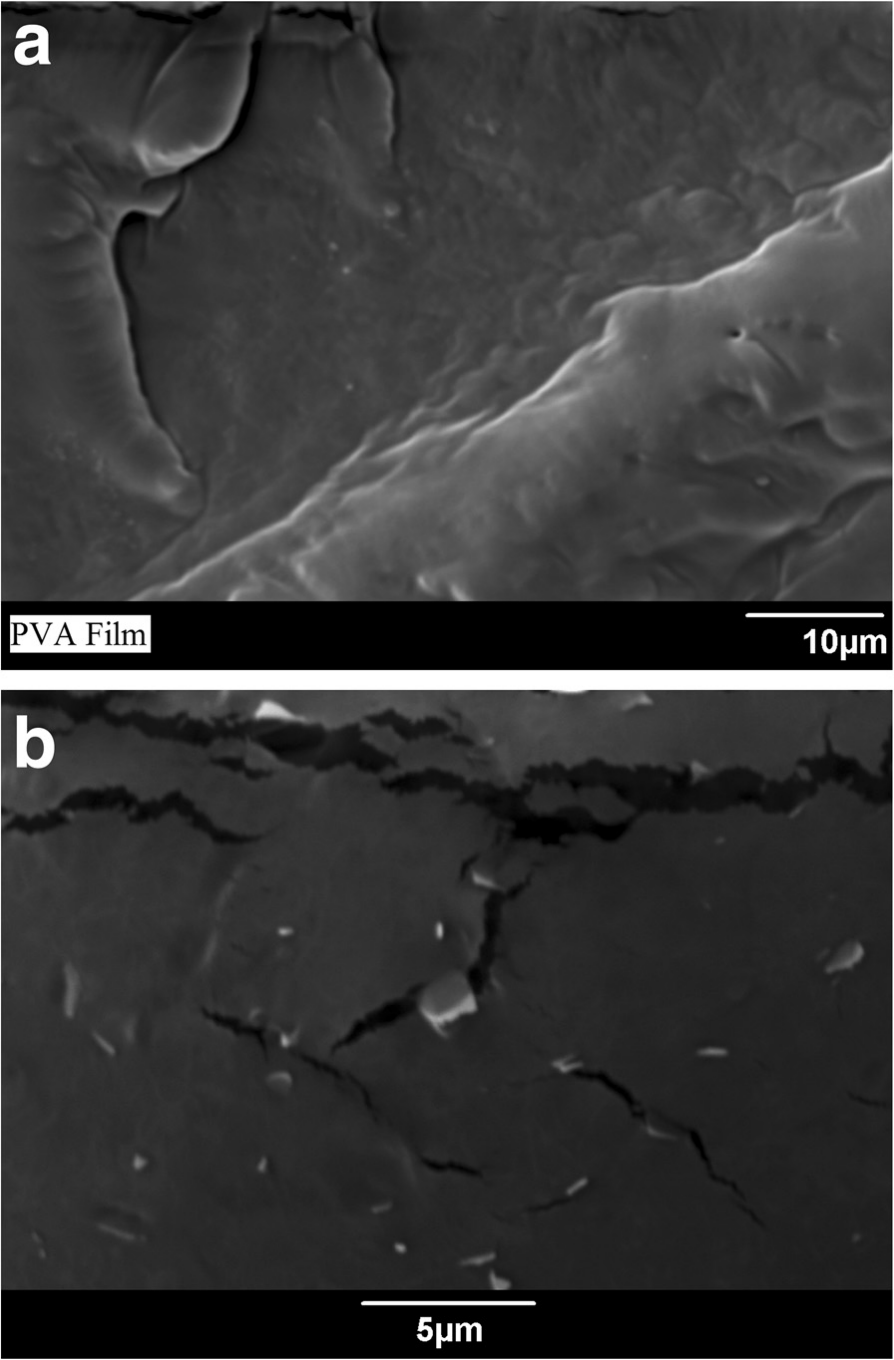
Scanning electron microscopy images of the fractured surfaces **a** base polymer film and **b** 0.006 *V*_*f*_ GNS-PVA composite

Atomic force microscopy (AFM) is also a very good tool to evaluate the lateral dimensions and number of layers of the graphene nanosheets. Few drops of the GNS/deionized water are dropped onto the silicon substrate and scanned by utilizing AFM (Jeol SPM 5200) in tapping mode. The representative atomic force micrograph presented in Fig. 2b give a clear indication of the GNS lateral dimension. It is confirmed from AFM image that the size of the GNS is around 1 μm. GNS prepared via liquid phase exfoliation are poly-disperse in size but the centrifugation process makes possible to select a range of sizes. The number of layers per sheets are 3~10 in general via liquid phase exfoliation method which is reasonable for the mechanical characteristics of PNCs [6, 28]. Interestingly, Gong et al. showed that the mechanical properties are maximized at *N* ~ 3 and monolayers are not essential for high degree of reinforcement [23]. Here in this case, AFM analysis predicts GNS thickness in the range 10–16 nm. It has been shown that for polymer-stabilized GNS, the monolayer thickness is ~2 nm [29]. Considering the polymer grafting on to the GNS, the number of GNS layers in our case is in the range *N* ~ 6–8 supporting the Raman spectra estimation of *N* ~ 8. GNS-PVA composites are prepared via the solution processing technique. It is assumed that with solution casting, the GNS are aligned in-plane inside nanocomposites. The final composites are in the form of thin films with approximate dimensions 3 × 3 cm. The thickness of the composite films is around 0.10–0.15 mm. These films are cut into strips (punched) for tensile testing. The dispersion of the GNS in nanocomposites is assessed via scanning electron microscopy of fractured surfaces. The samples were freeze-fractured in liquid nitrogen for the cross-sectional analysis. The micrograph shown in Fig. 3b predicts a homogeneous dispersion and no evidence of large aggregates at the maximum GNS loading (0.006 V_f_) while Fig. 3a is the neat polymer. The composites are characterized mechanically by tensile testing.

### Mechanical Characteristics Evaluation

**Fig. 4:**
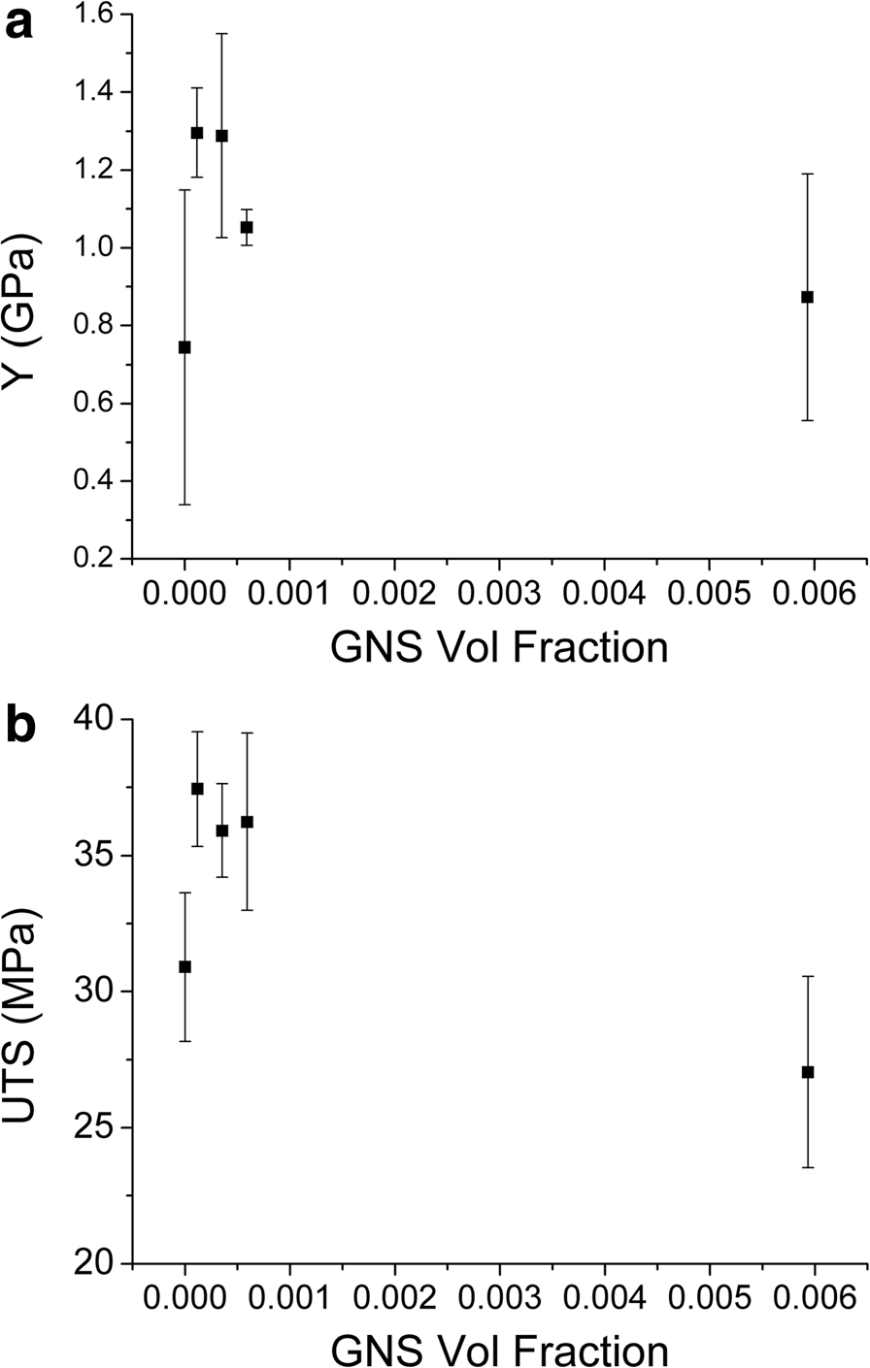
**a** Young’s modulus and **b** ultimate tensile strength of GNS-PVA nanocomposites as a function of GNS volume fraction

Young’s modulus (*Y*) of the nanocomposites is plotted as a function of GNS volume fraction (*V*_*f*_) shown in Fig. 4a. For each composite sample, five specimens are tested for mechanical property evaluation. The modulus increases linearly with GNS volume fraction up to a certain level and then declines a bit with further addition of GNS. Young’s modulus for neat PVA is around 0.74 GPa which reaches to the maximum value of around 1.3 GPa at 0.0001 *V*_*f*_ GNS concentration predicting a rate of increase of modulus, d*Y*/d*V*_*f*_ ~ 217 GPa. The ultimate tensile strength (UTS) of the composites is also measured and plotted as a function of GNS-*V*_*f*_ shown in Fig. 4b. The increase in strength is observed reaching ~20 % enhancement of the neat PVA by adding only 0.0001 *V*_*f*_ GNS. This increase in strength, when compared to the already reported results, is relatively on the lower side [5]. One reason may be that the dispersion of the GNS inside nanocomposites may vary with the increasing concentration. Generally, the Halpin-Tsai model is utilized for the composite modulus, *Y*, as a function of the filler volume fraction, *V*_*f*_, the moduli of filler, *Y*_*F*_, and matrix, *Y*_*M*_, as well as the nanosheet aspect ratio, *L*/*t*. According to this model [30]

3 $$ Y={Y}_M\left[\frac{1+2{V}_f\eta L/t}{1-{V}_f\eta}\right] $$

where

4 $$ \eta =\frac{Y_F/{Y}_M-1}{Y_F/{Y}_M+2L/t} $$

This particular model is suitable when nanosheets are aligned in the plane of the composite. In view of the low reinforcement level on the basis of misaligned GNS inside Krenchel’s approach for fiber-reinforced composites is considered along with the introduction of orientation parameter, *η*_0_. We can proceed by considering the following equation [13, 19]:

5 $$ Y\approx \frac{\eta_o{Y}_F{V}_f}{\left[\frac{Y_F/{Y}_M}{2L/t}+1\right]}+{Y}_M $$

Equation (5) is analogous to the modified rule of mixtures with a slight difference in the length efficiency factor [12]. The orientation parameter lies in the range 0 ≤ *η*_0_ ≤ 1. When the fillers are aligned parallel to the applied load, *η*_0_ = 1 and if the fillers are aligned perpendicular to the applied load, *η*_0_ = 0 [13, 31, 32]. Equation (5) is derived based on the approximation that *Y*_*F*_/*Y*_*M*_> > 1 and *V*_*f*_*η* < <1. Differentiating Eq. (5)

6 $$ \frac{dY}{d{V}_f}\approx \frac{\eta_o{Y}_F}{\left[\frac{Y_F/{Y}_M}{2L/t}+1\right]} $$

**Fig. 5:**
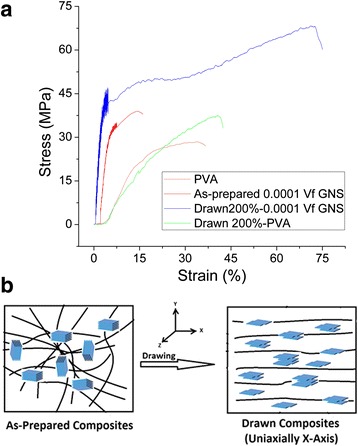
**a** Representative stress strain curves showing the effects of both, addition of GNS and drawing, on the mechanical properties of the PVA. **b** Schematic diagram for the as-prepared and uniaxially drawn composites showing clearly the effects of drawing in the drawn state as the polymer chains and few layer GNS are aligned along with the strain-induced exfoliation

**Fig. 6:**
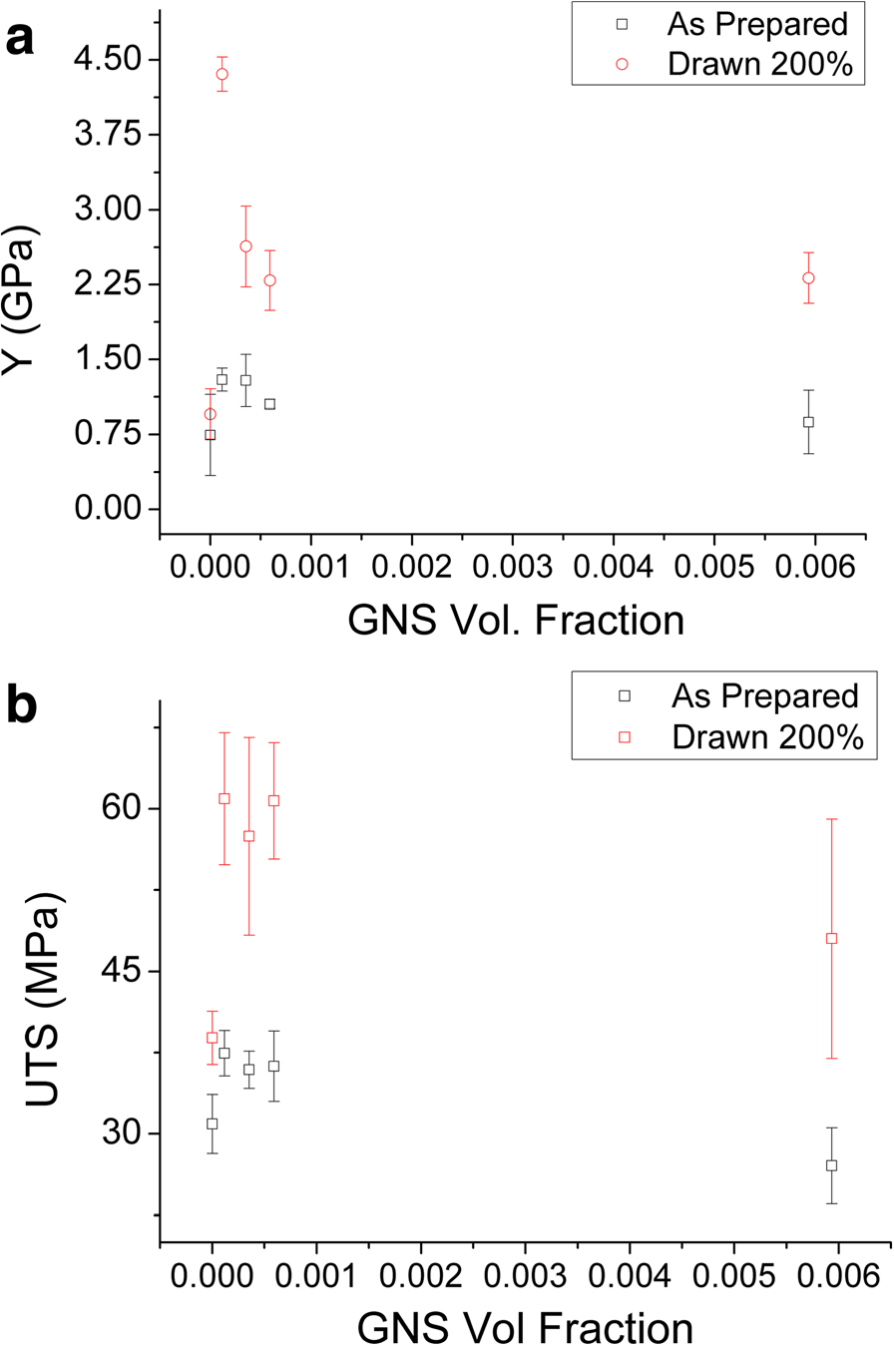
**a** The effect of drawing 200 % on the Young’s modulus and **b** ultimate tensile strength of GNS-PVA composites as a function of GNS volume fraction

It has recently been calculated that for randomly dispersed 2D nanosheets in PNCs, *η*_0_ is 0.38 [17]. Using *Y*_*F*_ ≈ 1000 GPa [28], *Y*_*M*_ ≈ 0.74 GPa, *L* ~ 1 μm and the thickness in the range of ~3 nm (as confirmed from Raman and AFM analysis), a good fit to the experimental data (d*Y*/d*V*_*f*_ ~217 GPa) is found when the orientation parameter *η*_0_ is fixed at 0.38~0.50. The data fits fairly well for *η*_0_ ~ 0.50, predicting that GNS are not fully aligned along the composite film plane. Thereby, Young’s modulus could be improved if the alignment of the GNS is enhanced. The well-established post-treatment technique of drawing is applied (experimental methods) and the composite films are drawn to 200 %. The composite films are marked before drawing and only those films are chosen for tensile tests which are drawn appropriately. The drawn composites are then divided (punched) in 3–5 strips to be able to evaluate the mechanical properties accurately. The drawn composite films are subjected to tensile test for the measurement of mechanical properties. All the samples were clamped carefully and made sure that they break around the center point. The samples broken near clamps were discarded. The representative stress strain curves are presented for the polymer and composites in both as-prepared and drawn states in Fig. 5a. Both “*Y*” and “UTS” are significantly increased for drawn composites with a very small amount of GNS addition to the polymer. The strain at break varied considerably from sample to sample, probably due to the combined effect of uniaxial drawing and aging, but generally remained below 100 %. Figure 5b shows the schematic model for the phenomena happening inside the composite after drawing. Figure 6 presents a detailed comparison of the mechanical characteristics for drawn and as-prepared composites. The modulus of neat PVA is increased from 0.74 to 0.95 GPa after drawing mainly due to the polymer chain alignment. More interestingly, “*Y*” at lower GNS loading is increased ~fourfolds for drawn composites at minute GNS loading. The rate of reinforcement “d*Y*/d*V*_*f*_” reaches to ~700 GPa for the drawn composites. Considering the GNS are fully aligned along drawn composite plane, the theoretical and experimental findings should fall in the same range. However, the theoretical results for d*Y*/d*V*_*f*_ are in the range of ~600 GPa for Y_F_ ~ 1000 GPa, Y_M_ ~ 0.95 GPa, L ~ 1 μm, t ~ 3 nm and the orientation parameter η_0_ ~ 1 (considering the perfect alignment after drawing) now. It is evident that the nanosheet alignment can increase the d*Y*/d*V*_*f*_ but not to the level achieved experimentally. This brings into account the already hypothesized claim of strain-induced exfoliation [19]. The delaminating nanosheets inside the matrix will decrease the thickness and by considering *t* ~ 1.5, the experimental and theoretical data coincides. Just as the case was with the BN-PVC composites [19], the shear delamination of the initially misaligned GNS is occurring due to which the thickness of the GNS is reduced ultimately. The UTS is also plotted as a function of GNS volume fraction both for as-prepared and drawn case in Fig. 6b. In the as-prepared case the enhancement in strength is only up to 20 % with respect to neat PVA which can be considered a marginal increment. However, drawing the composites to 200 % has significantly enhanced the strength both for neat PVA and the composites as well. The increase in polymer strength (30–38 MPa) is probably due to the polymer chain alignment. The enhancement in strength for drawn composites reaches up to 60 MPa which was around 38 MPa for as-prepared composites. For drawn composite case, the strength saturation shifts towards the right meaning that the strength enhancement is maintained for high GNS loading now. This could well be happening due to the shear delamination of the misaligned GNS due to the uniaxial drawing which must have acted as stress raisers in case of as-prepared composites [8]. To support the hypothetical argument of strain-induced exfoliation, well-established XRD technique is utilized for the analysis of the as-prepared and drawn samples of GNS-PVA composites.

### XRD Analysis

**Fig. 7:**
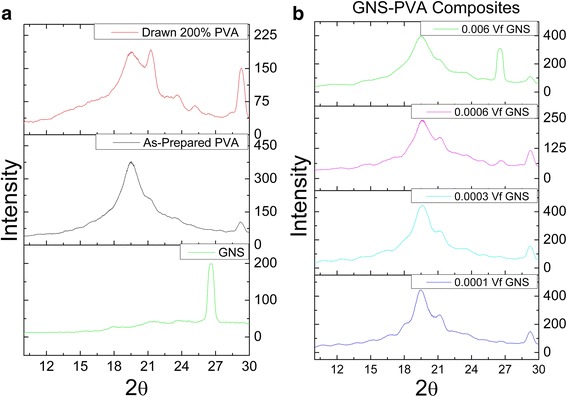
XRD patterns of **a** GNS, neat PVA, 200 % drawn PVA, **b** GNS-PVA composites at various filler fractions

X-ray diffraction (XRD) study is carried out for determination of the crystalline phase of GNS-PVA composites. Powder X-ray diffractometer is used having CuKα (1.54060 A°) as a source of radiation operated at 40 mA and 40 kV at room temperature and 2-theta (θ) ~ 10°–30°. In the first case, X-ray diffraction patterns have been utilized for studying the effects of polymer-stabilized GNS exfoliation and dispersion in as-prepared composites. The XRD plots for GNS, base polymer film, and drawn polymer film are shown in Fig. 7a while the GNS-PVA composite XRD results are presented in Fig. 7b. GNS diffraction peak appears at 2θ ~ 26.6°. The detailed analysis of the PVA shows its semicrystalline nature with the main peak position appearing at 2θ ~ 19.50° and the other smaller peak at 29.28°. The main crystalline peak at 19.50° with the d-spacing of 4.55 A° is assigned to the strong intermolecular interface linking of the PVA chains via intermolecular hydrogen bonding. The other minor peak appearing at 29.28° has the d-spacing in the range of 2 ~ 4 A° due to the hexagonal ordering inside polymer [33, 34]. Drawing 200 %, the main peak splits into two with another PVA peak appearing at 2θ ~ 21°. Also, an enhancement in the diffraction peak intensity at 2θ ~ 29° shows a clear trend of chain alignment due to uniaxial drawing. The XRD pattern for as-prepared GNS-PVA composites is shown in Fig. 7b. It shows that no GNS peaks are observed up until 0.0006 *V*_*f*_ GNS, confirming that GNS are very well dispersed and fully exfoliated. Furthermore, the diffraction peak intensity of PVA at 2θ ~ 19° for GNS-PVA is maintained with GNS, clearly showing that the crystallinity of the polymer is unaffected with the addition of GNS. At 0.0006 *V*_*f*_ GNS, there appears a tiny diffraction peak at 2θ ~ 26.6° which is enhanced at maximum loading of 0.006 *V*_*f*_ GNS. Once it is known that the GNS diffraction peak tends to appear at 0.0006 *V*_*f*_ GNS, the effect of drawing on the composites can be assessed at this loading and beyond to confirm the GNS delamination effect.

**Fig. 8:**
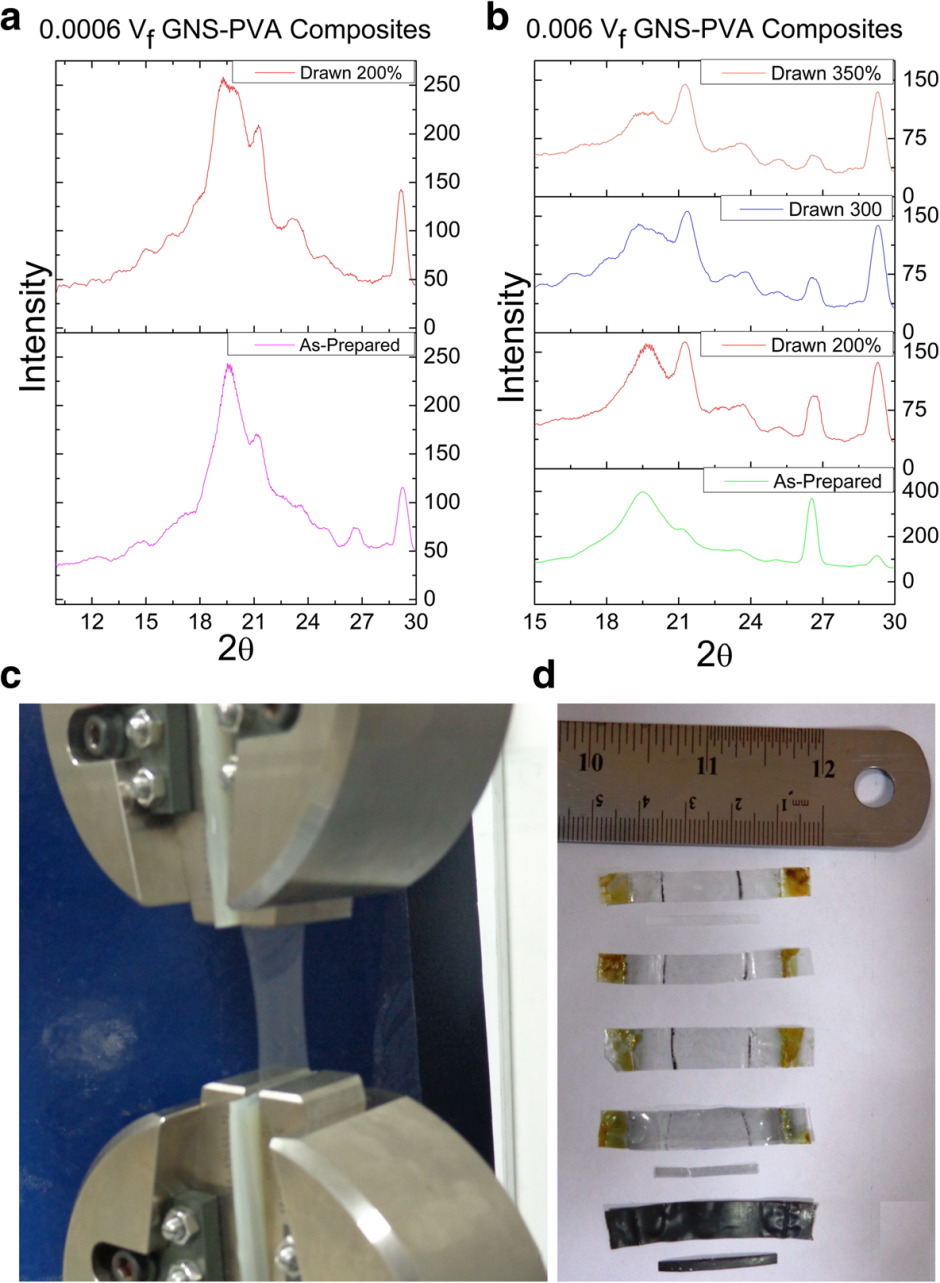
**a** Effect of drawing on the structural properties of 0.0006 *V*_*f*_ GNS-PVA at 200 % drawing in comparison with as-prepared composite. **b** The effect of drawing on the structural properties of 0.006 *V*_*f*_ GNS-PVA composites drawing at 200, 300, and 350 % in comparison with as-prepared composites. **c** Drawing process of 0.0006 *V*_*f*_ GNS-PVA via tensile tester. **d** As-prepared composite strips and drawn composite films for various GNS loading. The dimensions of as-prepared composite films are 30 × 30 mm^2^; half the film (15 × 30 mm^2^) is cut into various strips (2.25 × 30 mm^2^) while the other half is drawn 200 % and later cut into strips for tensile testing

Figure 8a gives a comparative analysis of the drawing effect on the GNS diffraction peaks for 0.0006 *V*_*f*_ GNS-PVA composites. It can be clearly seen that GNS diffraction peak completely disappears when the composites are uniaxially drawn 200 %. Drawing has not only induced the chain alignment but also improved the dispersion of GNS by delamination of the sheets in the composite system. Thereby, the significant improvement in the mechanical properties of drawn GNS-PVA composites is pertaining to such strain-induced delamination/exfoliation. The disappearance of the GNS diffraction peak with drawing 200 % drives us further to evaluate the composites with maximum GNS loading (0.006 V_f_). The diffraction peak intensity for the GNS peak lowers considerably in the first case with 200 % drawing as shown in Fig. 8b. In the next phase, the 0.006 *V*_*f*_ GNS-PVA composites are subjected to drawing 300~350 % as the optimum mechanical characteristics have been reported up to such strains and further strain cause breakage in polymer chains [18, 19]. The intensity of the GNS diffraction peak intensity is decreased noticeably and at 350 % drawing, it is lowered almost to the level of as-prepared 0.0006 *V*_*f*_ GNS-PVA composite case. The XRD analysis shows that drawing the composites induced the alignment of the GNS in the first place. On the other hand, the huge decrease in the intensity of GNS also confirms that with drawing, GNS have been delaminated and there are now lesser stacking layers available for the incoming x-rays.

Rao et al. [35] have also mentioned that the sharp reflection peak in the powder XRD pattern displays large number of layers and the reduction in this peak clearly suggesting the lesser number of graphene layers. Another characterization method which can be very useful in predicting the strain-induced exfoliation phenomena is Raman spectroscopy [36]. Although in present case, it was not possible as the spectra are dominated by the polymer because the graphene loading level is very low. It was very difficult to extract any information on the graphene in this case simply because of weak graphene signal. These measurements are tricky and require playing with acquisition parameters to be able to resolve the graphene while at the same time avoiding local heating. Theoretical predictions which are compromised in comparison with experimental results in the case of mechanical properties coincide only when the thickness of GNS is reduced. The delamination effect helps to exfoliate the GNS owing to the localized shearing imposed by drawing. Strain-induced exfoliation of GNS would remove the stress-concentration centers and will allow strength enhancement to persist to higher volume fractions as happened in the BN-PVC composite system [19]. This technique can be utilized further to improve the mechanical, thermal, and thermo-mechanical properties of graphene and other 2D material-based polymer nanocomposites.

## Conclusions

Composites of PVA filled with GNS are prepared. The relatively low levels of reinforcement are attributed to the non-aligned nature of GNS inside the PVA matrix. Drawing the composites to 200 % strain instigated the mechanical characteristics with a significant enhancement in both Young’s modulus (maximum ~×4) and ultimate tensile strength (maximum ~×2). The rate of increase in reinforcement cannot be explained solely on the basis of GNS alignment. Strain-induced exfoliation of GNS is suggested to play a role in improving the mechanical properties. To support the claim of strain-induced exfoliation, X-ray diffraction technique is utilized. The diffraction peak for graphene appears only around 0.0006 *V*_*f*_ GNS and at maximum loading 0.006 *V*_*f*_ GNS, graphene XRD peak intensity is high. These two particular loadings are subjected to drawing. The GNS diffraction peak for 0.0006 *V*_*f*_ completely disappears at 200 % drawing. The diffraction peak intensity of 0.006 *V*_*f*_ GNS-PVA composites at 350 % drawing is lowered to the level of as-prepared 0.0006 *V*_*f*_ GNS. It clearly shows that GNS layers are reduced inside the matrix. The strain-induced exfoliation along with the alignment of GNS is thought to be the reasons for the significant reinforcement achieved for GNS-PVA composites.

## Abbreviations

2D: two-dimensional
AFM: atomic force microscopy
FWHM: full width half maximum
GNS: graphene nanosheets
H_2_O: water
hBN: hexagonal boron nitride
PNCs: polymer nanocomposites
PVA: polyvinyl alcohol
PVC: polyvinyl chloride
rpm: rotation per minutes
UTS: ultimate tensile strength
XRD: X-ray diffraction
